# UC-BSCs Exosomes Regulate Th17/Treg Balance in Patients with Systemic Lupus Erythematosus via miR-19b/KLF13

**DOI:** 10.3390/cells11244123

**Published:** 2022-12-19

**Authors:** Jianxin Tu, Nan Zheng, Chentong Mao, Shan Liu, Hongxing Zhang, Li Sun

**Affiliations:** 1 Department of Rheumatology and Immunology, The First Affiliated Hospital of Wenzhou Medical University, Wenzhou 325000, China; 2 School of Pharmacy, Wenzhou Medical University, Wenzhou 325000, China

**Keywords:** systemic lupus erythematosus, umbilical cord blood mesenchymal stem cells, inflammatory factors, mathematical and statistical analysis, exosomes, peripheral blood mononuclear cells

## Abstract

Umbilical cord blood mesenchymal stem cells (UC-BSCs) are cells with low immunogenicity and differentiation potential, and the transfer of exosomes carried by UC-BSCs can regulate innate and adaptive immunity and affect immune homeostasis. This is an area of focus for autoimmune illnesses such as systemic lupus erythematosus (SLE). The target of this research was to investigate the immunomodulatory effect of exosomes produced from mesenchymal stem cells on SLE and its mechanism. After isolation of peripheral blood mononuclear cells (PBMC) from the SLE group and healthy group and treatment of SLE-derived PBMCs with UC-BSC-derived exosomes, the mRNA levels of corresponding factors in cells under different treatments were determined by RT-PCR, Th17/Treg content was analyzed by FCM (flow cytometry), and the targeted binding of microRNA-19b (miR-19b) to KLF13 was identified by in vitro experiments and bioinformatics analysis. The findings demonstrated that PBMC cells from SLE patients had higher proportions of Th17 subsets than the control group, whereas Treg subgroups with lower percentages were discovered. miR-19b’s expression level was markedly reduced, which was inversely associated to the concentration of KLF13. In vitro experiments show that UC-BSC-derived exosome treatment can target KLF13 expression by increasing the miR-19b level, thereby regulating Th17/Treg balance and inhibiting the expression of inflammatory factors. According to the study’s findings, SLE patients have dysregulated expression of the genes miR-19b and KLF13, and UC-BSC exosomes could regulate Th17/Treg cell balance and inflammatory factor expression in SLE patients through miR-19b/KLF13.

## 1. Introduction

Chronic autoimmune illness systemic lupus erythematosus (SLE) affects numerous organ systems. It usually occurs in women between adolescence and menopause [1,2]. SLE is an example of an autoimmune disease where epigenetic dysregulation—including DNA methylation, histone alterations, and noncoding RNAs—plays a significant role. Studies have found that miRNAs are abnormally expressed in patients with SLE, and can promote the progression of SLE [3,4].

Mesenchymal stem cells from umbilical cord blood (UC-MSCs) are pluripotent stem cells with the capacity to develop into a range of cell types. Because of their rich sources, strong differentiation potential, and easy access, they are widely used in therapy for various diseases [5,6]. Recent studies have pointed out [7,8,9] bone marrow mesenchymal stem cells contain differentially expressed miRNAs, and UC-MSC transplantation therapy or UC-MSC-derived exosome treatment can effectively treat a variety of malignant tumors, autoimmune diseases, fractures, and bone loss. Exosomes can move through the blood [8]. Some studies have shown [9,10] that exosomes can be used as extracellular vesicles to transfer miRNA, mRNA, and proteins to regulate cell activity. Chemokines are a class of small molecular proteins that mainly have chemotactic effects on leukocytes and other biological effects, and can significantly affect the regulation of inflammatory and defensive reactions [11]. However, the continuous high-level expression of chemokines will also increase the recruited leukocytes and cause destructive damage, which is crucial to tissue and organ damage. The chemotactic cytokine CC subfamily includes the regulated upon activation typical T cell produced and released factor (SLENTES), which is highly expressed in SLE and is closely related to tissue and organ inflammatory damage, when T cells are stimulated late, a transcription factor called KLF13 (SLENTES factor of late-activated T lymphocytes-1, RFLAT-1) encourages the production of SLENTES [11,12]. In this study, we explored the relationship between miR-19b and KLF13 expression in the peripheral blood of SLE patients and the role of UC-MSC-derived exosomes in regulating Th17/Treg homeostasis, in order to provide new ideas for the treatment of SLE.

## 2. Methods and Reagents

### 2.1. Research Object

A total of 47 patients with SLE were all outpatients at the Affiliated Hospital of Wenzhou Medical University’s Department of Rheumatology and Immunology. The diagnosis of SLE met at least 4 of the 11 criteria recommended by the American College of Rheumatology for SLE classification. There were 15 healthy volunteers in the control group. Between the two groups, there were no discernible differences in age or gender. The Hospital Ethics Committee gave the study their blessing.

### 2.2. Cell Sorting and Culture

Human umbilical cord blood stem cells and CD4+ T cells were obtained from the Cell Resource Center of Chinese Academy of Sciences. By using the density gradient approach, peripheral blood mononuclear cells (PBMCs) were extracted (Ficoll), and human umbilical cord blood stem cells (UC-BSCs) were obtained from Wenzhou Youren Cell Biology Company, and cultured in DMEM medium (Gibco, Thermo Fisher Scientific, Waltham, MA, USA) (Invitrogen, Thermo Fisher Scientific, Waltham, MA, USA) at 37 °C and 5% CO_2_.

### 2.3. Exosome Isolation

Exosomes were harvested by ultracentrifugation: cultured in medium for 50 h, harvest cells and centrifuged at 125,000× *g* for 8 h. The supernatant was then centrifuged at 900× *g* for 25 min, 15 min at 1500× *g*, 15 min at 5000× *g*, and 30 min at 12,000× *g*. The supernatant was converged after centrifugation, and the epipelagic was ultracentrifuged at 125,000× *g* for 60 min. The exosomes were then washed and pelleted in the same manner.

### 2.4. Exosome Characterization

To disperse the exosomes equally, 20 μg of them were melted in PBS and shaken for a minute. Under the help of a NanoSight nanoparticle tracking analyzer scale distribution, the exosomes were then quantified and examined. The exosomes were rapidly fixed for two hours at four degrees by glutaraldehyde and then using PBS to wash exosomes. Then the exosomes were dewatered after fixation, macerated by epoxy resin, sliced to 0.5 μm thick, and stained with uranyl acetate and lead citrate after light microscopy positioning, and observed under microscopy.

### 2.5. Cellular Uptake of Exosomes

The exosomes were collected by centrifugation (125,000× *g*, 60 min) and incubated with CFSE dye for 10 min at room temperature, CFSE-labeled exosomes were added to CD4+ cell culture medium and incubated at 37 °C for 6, 12, 18, 24, 36, and 48 h, and cellular uptake was scanned by fluorescence microscopy.

### 2.6. Detection of Th17/Treg Cell Content

Th17 cell detection was performed: 100 μL Th17 permeable wash buffer A was incubated at room temperature in the dark for 10 min, then an appropriate amount of PBS was added. It was centrifuged at 2000 rpm for 5 min, then the supernatant was removed and 100 μL was added. Next, fluorescently labeled anti-IL-17 antibody was added, it was left to stand in the dark environment for 20 min, cells were resuspended with the appropriate amount of PBS, then transferred to a flow tube and analyzed on the machine. Treg cell detection had the following process: 500 μL of Foxp3 permeabilization wash buffer was incubated at 4 °C in the dark for 25 min, then washed with PBS and centrifuged at 2000 rpm for 10 min. Then, the supernatant was removed and 5 μL of fluorescence labeled anti-Foxp3 antibody was added. It was placed it in the dark for 30 min, the cells were resuspended with PBS, transferred to EP tubes, and identified by flow analysis. In addition, the effect of exosomes on Th17/Treg cell homeostasis and inflammatory factors was studied by adding or not adding 5 μM of exosomes to isolated PBMC and incubating for 48 h with reference to the above-mentioned Th17/Treg cell content assay.

### 2.7. RT-PCR

Total RNA of peripheral blood mononuclear cells was extracted using the Trizol solution, and A260/A230 ratio was detected using a Thermo Scientific™ NanoDrop™ 2000 microspectrophotometer (NanoDrop, Waltham, MA, USA). TaqMan MicroRNA Reverse Transcription Kit was used to get cDNA, then cDNA was diluted to 50 ng/L, and then detected by real-time quantitative PCR using ABI 7300 (Table 1).

### 2.8. Cell Transfection

According to the instructions of interferin siRNA transfection reagent (polyplus company), transfection of NC mimics or miR-19b mimics into CD4+ T cells was performed. On the 3rd and 5th day, fresh culture medium (DMEM nutrient solution containing 10% FBS) was added, and the cell density was maintained at 106/mL in the cultured cells. The primer sequences used were synthesized by Zhejiang Gloucester Biotechnology Company (Zhejiang, China).

### 2.9. Dual Luciferase Reporter Gene Assay

The wild-type and 3’UTR mutant sequences of KLF13 containing possible miR-19b binding locations were put into pMIR-REPORT™ luciferase vector (Nantong Yishi Biotechnology Co., Ltd., Nantong, Jiangsu, China) using Spe I and Hind III restriction sites, Jiangsu China), the plasmids were named Est-1-WT and Est-1-MUT. After utilizing Lipofectamine 3000 to co-transfect KLF13-WT, KLF13-MUT, and pMIR Vector with miR-19b mock or negative control, cells were collected, respectively. The Dual-Glo Luciferase Reporter Kit was used to quantify luciferase activity after 50 h. The ratio of RLU1/RLU2 was then used to express the relative fluorescence intensity ratio. Each construct’s normalized firefly luciferase activity was compared to the pMIR Vector no-insert control’s.

### 2.10. Statistical Analysis

Statistical analysis was implemented using SPSS 23.0 software, IBM, New York, NY, USA. Normal distribution test and homogeneity of variance were performed, and normal distribution data were stated as mean ± SD. Statistics between two groups were compared using an independent sample *t*-test, while data between several groups were compared using a one-way ANOVA. Non-normally distributed data were tested by non-parametric test, and *p* < 0.05 was used to define statistical significance.

## 3. Results

### 3.1. Comparison of miR-19b Expression Levels

**Figure 1:**
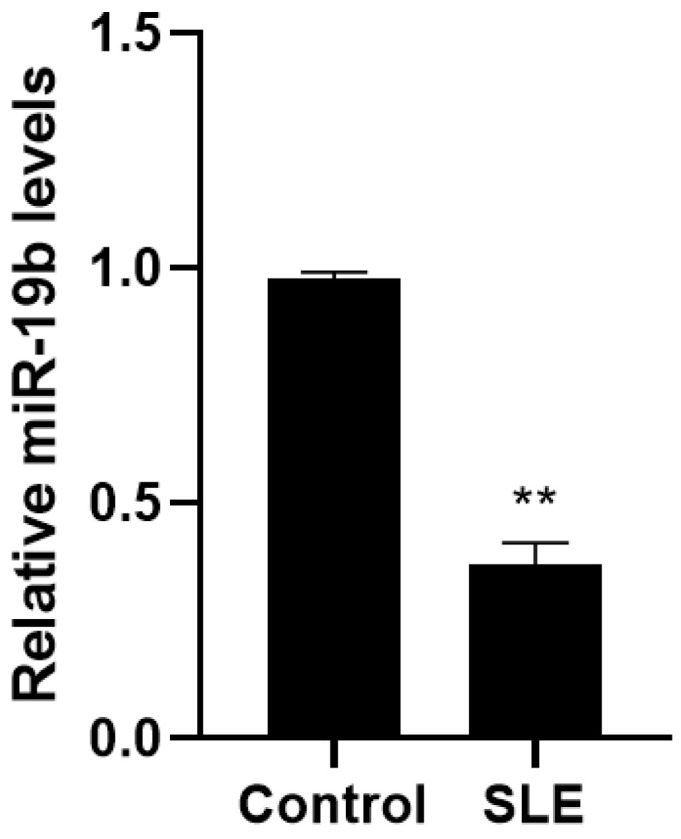
Expression degrees of miR-19b in SLE patients and normal controls. (Compared with the control group, ** *p* < 0.01).

The expression levels of miR-19b in PBMCs of the two study groups were analyzed by qRT-PCR, in which the expression levels of miR-19b in PBMCs of SLE patients were significantly lower than those of normal controls (*p* < 0.05, Figure 1), and the differences were statistically significant (*p* < 0.05).

### 3.2. Balance of Th17 and Treg Cell Subsets

**Figure 2:**
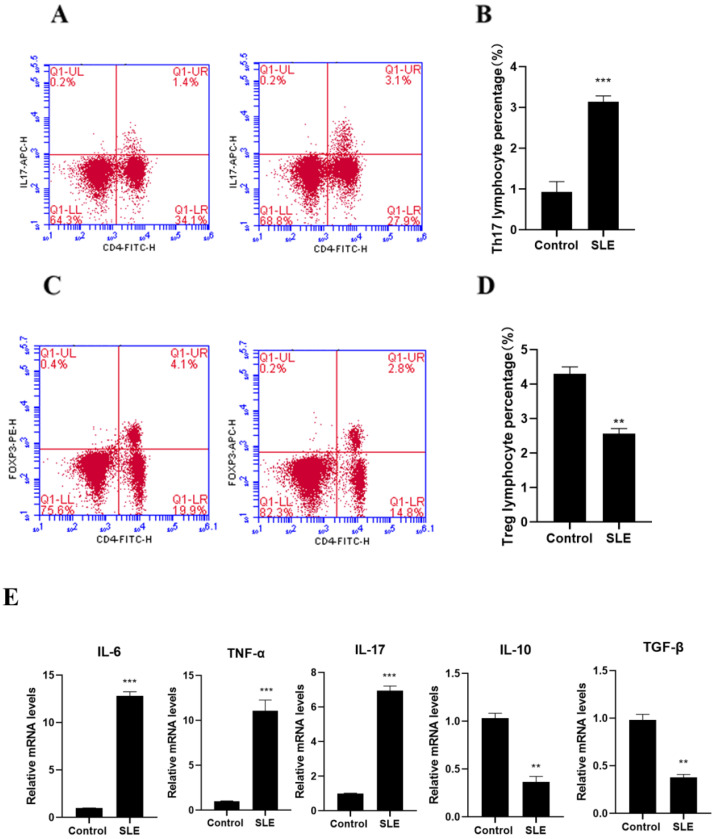
Imbalance of Th17/Treg production in SLE patients. (**A**) Flow analysis of CD4 + IL-17 + Th17 cells, (**B**) percentage of CD4 + IL-17 + Th17 cells, (**C**) flow analysis of CD4 + Foxp3 + Treg cells, (**D**) CD4 + Foxp3 + Treg percentage of cells, (**E**) PBMC production of cytokines IL-6, TNF-α, IL-17, IL-10, and TGF-β, compared to control. ** *p* < 0.01, *** *p* < 0.001.

Flow cytometry showed that compared with control cells, the proportion of Th17 subsets in SLE patient-derived PBMC cells increased, while the proportion of Treg subsets decreased (Figure 2A–D). Compared with the control group, the expressions of TNF-α, IL-6, and IL-17 in PBMC of SLE patients were significantly increased, while the expression of IL-10 and TGF-β was dramatically reduced. (*p* < 0.05, Figure 2E).

### 3.3. Identification of miR-19b Target Genes

**Figure 3:**
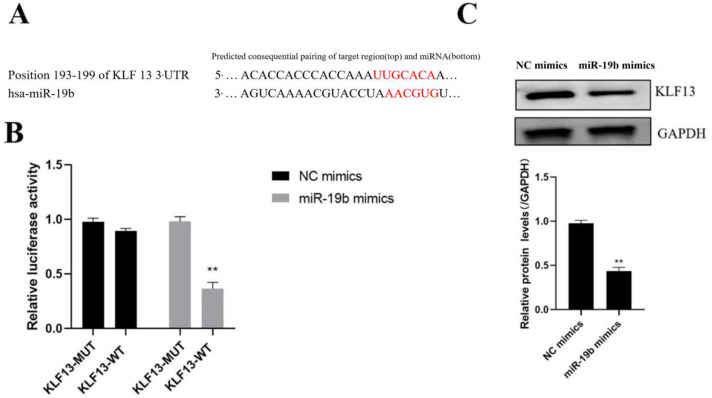
miR-19b targets KLF13 expression. (**A**) Bioinformatics website analysis of the binding site of KLF13 3’-UTR to miR-19b, (**B**) dual luciferase reporter gene to identify miR-19b and KLF13 binding, (**C**) Western blot analysis of several transfections protein expression of post-KLF13. Compared with NC mimics group. ** *p* < 0.01.

Target Scan, a bioinformatics program, was used to estimate the miR-19b target genes, which showed that KLF13’s 3’UTR contained a miR-19b binding sequence (Figure 3A). Further dual-luciferase reporter gene and Western blotting analysis indicated that the miR-19b overexpression vector can significantly down-regulate luciferase activity and KLF13 protein level (*p* < 0.05, Figure 3B,C).

### 3.4. Relationship of KLF13 and miR-19b Expression in SLE Patients

**Figure 4:**
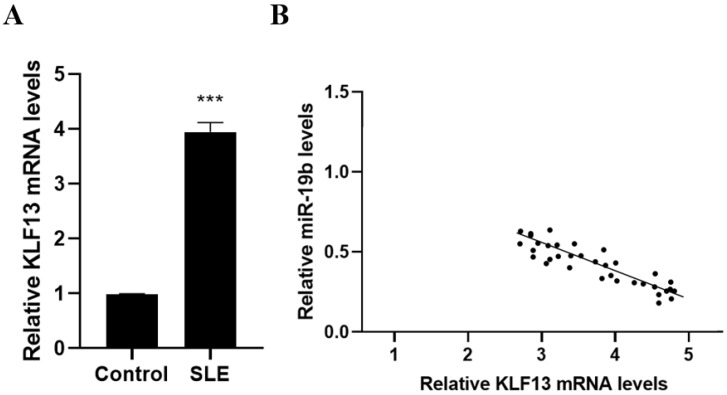
Inverse correlation between KLF13 and miR-19b expression in SLE patients. (**A**) The mRNA expression of KLF13 in SLE patients, (**B**) connection between KLF13 expression level and miR-19b expression level in SLE group, compared with the control group. *** *p* < 0.001.

The expression of KLF13 in SLE patients was determined using RT-PCR, and the outcomes indicated that the mRNA level of KLF13 in SLE patients was a lot higher than it was in the typical controls (*p* < 0.05, Figure 4A). As a result, the expression of KLF13 and miR-19b were inversely linked (r = −0.318, *p* < 0.05, Figure 4B).

### 3.5. Uptake of UC-MSC Exosomes by PBMC Cells

**Figure 5:**
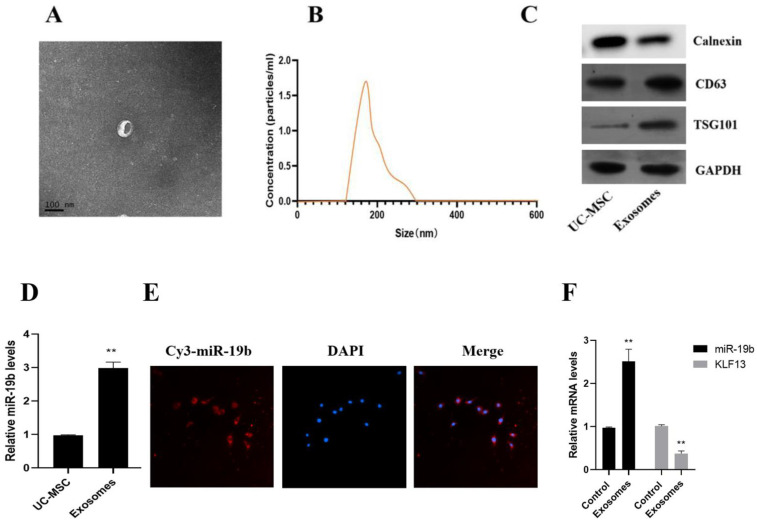
Isolation and characterization of exosomes in UC-MSCs. (**A**) Transmission electron microscope image of exosome morphology (scale bar: 100 μm), (**B**) Size distribution of exosomes, (**C**) CD63 and TSG101 expression, (**D**) UC-MSC exosomes expression of miR-19b in exosomes, (**E**) uptake of exosomes by PBMC cells (scale bar = 25 μm) (**F**) the expression of KLF13 and miR-19 in PBMC cells after UC-MSC exosome treatment for 48 h compared to the control group. ** *p* < 0.01.

UC-MSC exosomes were rotund or oval with sizes ranging from 30–100 nm and with a low-density content of intact membrane components after differential ultracentrifugation to separate exosomes (Figure 5A). The average size of cellular exosomes, according to nanoparticle tracking analysis, was 132.5 ± 37.4 nm. We also found that the concentration of exosomes was 1.7 × 10^11^/L (Figure 5B). Furthermore, the outcomes of Western blotting showed that the isolated exosomes expressed CD63 and TSG101, two exosome-representative markers, while lacking cadherin, an exosome-nonrepresentative marker. These results demonstrate the successful isolation of MSC (UC-MSC)-derived exosomes. The qRT-PCR results revealed that purified exosomes expressed more miR-19b than donor UC-MSCs (Figure 5D). In conclusion, exosomes in UC-MSCs can enrich miR-19b.

Microscopic observations revealed that miR-19b in UC-MSCs could be efficiently transduced into CD4+ cells via exosomes. Exosomes that were CFSE-labeled were found in the cytoplasm, and UC-MSC-derived miR-19b was successfully transduced into CD4+ cells (Figure 5E). After 48 h of incubation with CFSE exosomes, high levels of miR-19b expression in CD4+ cells were found (Figure 5F).

### 3.6. Effects of UC-MSC Exosomes on Th17/Treg

**Figure 6:**
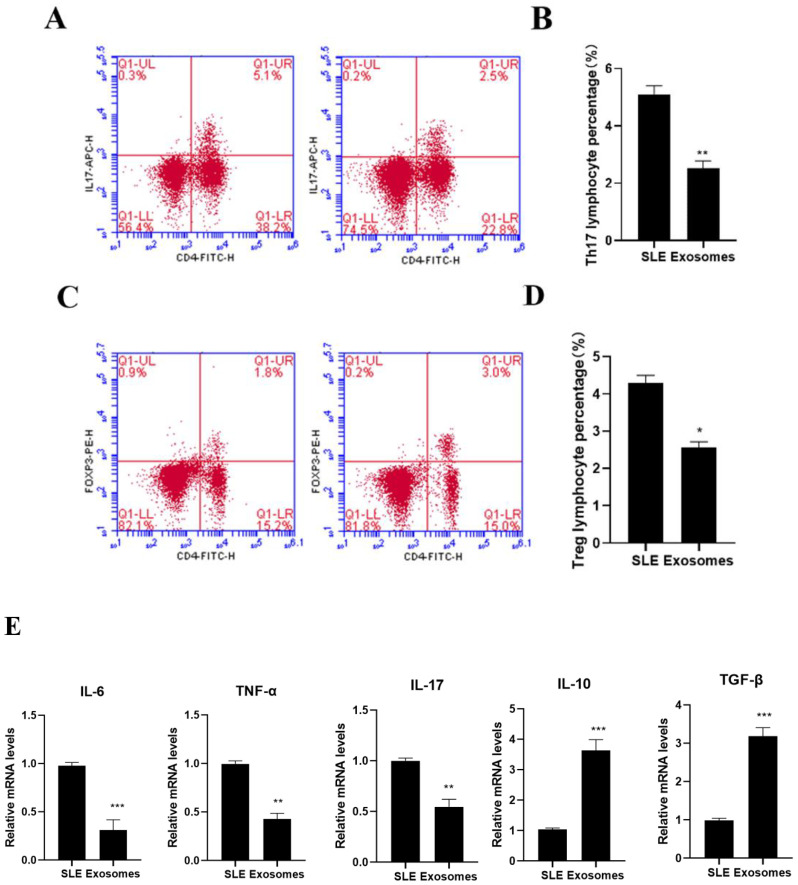
Impact of UC-MSC exosomes on Th17/Treg. (**A**) Flow analysis of CD4 + IL-17 + Th17 cells, (**B**) percentage of CD4 + IL-17 + Th17 cells, (**C**) flow analysis of CD4 + Foxp3 + Treg cells, (**D**) CD4 + Foxp3 + percentage of Treg cells, (**E**) PBMC production of cytokines IL-6, TNF-α, IL-17, IL-10, and TGF-β, compared with SLE group. * *p* < 0.05, ** *p* < 0.01, *** *p* < 0.001.

Exosome group cells had a lower percentage of Th17 subsets than cells from the SLE group, while their percentage of Treg subsets increased, according to flow cytometry (Figure 6A–D). The exosomes group’s TNF, IL-6, and IL-17 expression levels were considerably lower than those of the cells in SLE patients, while those of IL-10 and TGF- were significantly higher, according to RT-PCR detection. (*p* < 0.05, Figure 6E).

## 4. Discussion

A miRNA called miR-19b is widely distributed in the human body and is expressed in many cells. It can play a physiological role by regulating cell differentiation, energy metabolism, migration, etc. [11]. Research reports in recent years have found that miR-19b is associated with disease progression, is misexpressed in many different human diseases such as atherosclerosis and diabetes, and is involved in the regulation of disease progression [13,14,15]. In this study, we first eliminated SLE patients with abnormally low miR-19b expression. Using bioinformatics methods, we hypothesized that miR-19b’s target gene is KLF13, the primary transcription factor that secretes RANTES during the latter stages of T lymphocyte activation. RANTES is a typical C-C subfamily member chemotactic cytokine that can cause T cells, eosinophils, and other cells to infiltrate the site of inflammation [11,12]. According to the study’s findings, there was a significant inverse relationship between the expression of miR-19b and KLF13 in SLE patients. Further in vitro experiments showed that miR-19b could inhibit the expression of endogenous KLF13 in T cells. The luciferase reporter gene experiments also demonstrated direct miR-19b binding to the 3’UTR of the KLF13 mRNA, suggesting the connection between the high expression of KLF13 and the dysregulated miR-19b expression in SLE patients.

In SLE, inflammatory cytokines play a major role, and Treg and Th17 cell subgroups can mediate the secretion of inflammatory cytokines [13]. Recent research found [14,15,16], in the stage of active SLE, Treg levels are low and Treg/Th17 levels are decreasing. In line with this, flow cytometry results from this study revealed that the proportion of Th17 subsets in T cells derived from SLE patients was increased while the proportion of Treg subsets was reduced. The levels of IL-6, IL-21, and TGF- are connected to the differentiation and maturation of Th17 cells, and serum IL-17 is a marker of Treg cells [17,18,19]. In this study, RT-PCR detection showed that, in comparison to T cells derived from healthy controls, T cells in SLE patients had considerably greater expressions of IL-6, IL-17, and TNF, as well as significantly higher expressions of IL-10 and TGF-. The expression level was significantly lower, indicating that SLE patients have autoimmune and inflammatory responses.

MSCs are a successful method for treating SLE and can significantly lower the disease’s harmful autoimmune and inflammatory responses [20,21,22]. Recent literature also points out [23,24] that co-culture of UC-MSCs can prevent the differentiation of naive T cells into Th17 cells and treat persons with juvenile idiopathic arthritis (JIA) by downregulating the level of TNF-αand IL-6. Exosomes from UC-MSCs were first identified and detected in this study, and the results revealed that miR-19b expression was dramatically higher in exosomes derived from UC-MSCs than in UC-MSC cells. When UC-MSC-derived exosomes were used to treat SLE patients, compared to untreated SLE patient-derived T cells, the ratio of Th17 subsets decreased, while the ratio of Treg subsets increased. Additionally, the expressions of IL-6, IL-17, and TNF- were dramatically reduced, while those of IL-10 and TGF- were dramatically increased. However, the shortcoming of this study is that we did not demonstrate the detailed mechanism by which miR-19b and KLF13 regulate Th17/Treg homeostasis, and this issue will be addressed in our next studies.

In summary, our findings demonstrated that miR-19b expression was decreased in SLE patients compared to controls, whereas KLF13 expression was elevated. It was confirmed using in vitro experiments that UC-MSC-derived exosomes can promote T cells to express miR-19b, which inhibits the level of KLF13, regulates the balance of Th17/Treg cells, and inhibits the production of inflammatory cytokines, thereby exerting immunosuppressive and inflammatory inhibitory effects.

## Figures and Tables

**Table 1:** Sequences of primers used.

Gene	Forward (5′-3′)	Reverse (5′-3′)
IL-6	CCG GTT CTG TTC TGC CAG CG	CTG CGA CAC TCA CCT CTG CA
IL-17	CTC CTG TGA TGG CCC ACC TG	CTC CTG GAT CTG AGA CGG AT
IL-10	CGG TCA AGA TCG CCA CTG	ACG GCT GAC TCT AGT AGG GC
TGF-β	AAC GAA CTG GCT AGG TGC	CCT AGG CTC ATT AGG CTT AG
MiR-19b	CAA GTG GAA GTG GGC AGA G	
GAPDH	CAAGAGCCAACGAGCCAAGTC	GCCCAACGTGGACTCCAATTGG

## Data Availability

Data available on request from the authors.
